# Guillain–Barre syndrome outbreak in Peru: Association with polymorphisms in *IL‐17*, *ICAM1*, and *CD1*

**DOI:** 10.1002/mgg3.960

**Published:** 2019-08-28

**Authors:** Luis Jaramillo‐Valverde, Kelly S. Levano, Isolina Villanueva, Meylin Hidalgo, Marco Cornejo, Pilar Mazzetti, Mario Cornejo‐Olivas, Cesar Sanchez, Julio A. Poterico, Julio Valdivia‐Silva, Heinner Guio

**Affiliations:** ^1^ INBIOMEDIC Research and Technological Center Lima Peru; ^2^ ALBIOTEC Lima Peru; ^3^ School of Public Health and Administration Universidad Peruana Cayetano Heredia Lima Peru; ^4^ Hospital Belén de Trujillo La Libertad Peru; ^5^ Neurogenetics Research Center Instituto Nacional de Ciencias Neurológicas Lima Peru; ^6^ School of Medicine Universidad Nacional Mayor de San Marcos Lima Peru; ^7^ Center for Global Health Universidad Peruana Cayetano Heredia Lima Peru; ^8^ Servicio de Genética Instituto Nacional de Salud del Niño San Borja (INSN‐SB) Lima Peru; ^9^ Department of Bioengineering and Chemical Engineering Universidad de Ingenieria y Tecnologia ‐ UTEC Lima Peru; ^10^ Universidad Científica del Sur Lima Peru; ^11^ Universidad de Huánuco Huánuco Peru

**Keywords:** Guillain-Barre syndrome, *CD1*, genetic polymorphism, *ICAM1*, *IL‐17*

## Abstract

**Background:**

Guillain–Barre Syndrome (GBS) is considered a complex disorder with significant environmental effect and genetic susceptibility. Genetic polymorphisms in *CD1E*, *CD1A*, *IL‐17*, and/or *ICAM1* had been proposed as susceptibility genetic variants for GBS mainly in Caucasian population. This study explores the association between selected polymorphisms in these genes and GBS susceptibility in confirmed GBS cases reported in mestizo population from northern Peru during the most recent GBS outbreak of May 2018.

**Methods:**

A total of nine nonrelated cases and 11 controls were sequenced for the polymorphic regions of *CD1A*, *CD1E*, *IL‐17*, and *ICAM1*.

**Results:**

We found a significant protective association between heterozygous GA genotype in *ICAM1* (241Gly/Arg) and GBS (*p* < .047). *IL‐17* was monomorphic in both controls and patients. No significant differences were found in the frequency of SNPs in *CD1A* and *CD1E* between the group with GBS patients and healthy controls.

**Conclusion:**

I*CAM1* polymorphisms might be considered as potential genetic markers of GBS susceptibility**.** Further studies with larger sample size will be required to validate these findings.

## 1 INTRODUCTION

Guillain–Barre syndrome (GBS) is an acute inflammatory polyradiculoneuropathy with ascending weakness staring in lower limbs, extension to the upper limbs and face, as well as complete loss of deep tendon reflexes (Esposito & Longo, 2017; Rodríguez et al., 2018; Wijdicks & Klein, 2017; Winner & Evans, 1990). The annual incidence of GBS is 0.5–2 cases per 100,000 people, which increases with age (Esposito & Longo, 2017; Winner & Evans, 1990). GBS is rare in children under 2 years (Rosen, 2012); males are 1.5 times more likely to suffer GBS than women (Esposito & Longo, 2017; Hughes & Cornblath, 2005; Rosen, 2012). The exact cause of GBS has not been defined yet; however, 50%–70% of the cases appear 1–2 weeks after an infection (bacterial or viral) inducing an aberrant autoimmune response directed to the peripheral nerves and their spinal roots (Ropper, 1992; Esposito & Longo, 2017; Walgaard et al., 2011). GBS is known to occur in several forms including acute inflammatory demyelinating polyradiculoneuropathy (AIDP), Miller Fisher syndrome (MFS), acute motor axonal neuropathy (AMAN), and acute motor‐sensory axonal neuropathy (AMSAN).

The interaction between microbial and host factors has been poorly studied in GBS, as well as the genetic susceptibility of an individual to develop this syndrome. Possible markers of genetic susceptibility to GBS have been reported, including *CD1E* (OMIM #188411), *CD1A* (OMIM #188370), *IL‐17* (OMIM #606496), and *ICAM1* (OMIM #147840) (Caporale et al., 2006; Kharwar, Prasad, Singh, Paliwal, & Modi, 2017). *CD1E* and *CD1A* are glycoproteins of major histocompatibility complex (MHC) specialized in capturing and presenting glycolipids to T cells (Caporale et al., 2006; Porcelli & Modlin, 1999). In a research study conducted by Caporale et al, it was reported that individuals with the genotype CD1E*01/01 were 2.5 times more susceptible to develop GBS, while individuals with the genotypes CD1A*01/02 or CD1E*01/02 had a risk of 3.6 and 2.3 times lower, respectively (Caporale et al., 2006). Likewise, there has also been an association of GBS with polymorphisms *IL‐17* (Glu126Gly) and *ICAM1* (Gly241Arg) (Kharwar et al., 2017). *IL‐17* regulates the expression of inflammatory genes, including proinflammatory chemokines, hematopoietic cytokines, acute phase response genes, and antimicrobials (Shen & Gaffen, 2008) in neutrophils, macrophages, and endothelial cells (Zepp, Wu, & Li, 2011). On the other hand, previous studies show that *ICAM1* plays a central role in the development of demyelinating disease (Musso et al., 1994).

GBS and its association with a variety of infectious agents have been reported in Peruvian population. By 2014, case series of 32 GBS cases followed in Lima (capital city) found that AIDP was the most common form (75%) followed by AMAN and MFS with frequencies of 18.8% and 6.3%, respectively (Apaza Nina, 2014). By contrast, series from northern Peru (2017) found 16 Peruvian cases where AMSAN was the most common form (37.5%) followed by AMAN (25%) and AIDP (12.5%) (Ballón‐Manrique & Campos‐Ramos, 2017). In 1987, five GBS cases were associated with a viral infection caused by a rabies vaccine prepared with the brain of a lactating mouse (Cabrera, Griffin, & Johnson, 1987). In 2010, a GBS case was reported associated to Brucellosis, an infectious disease caused by Brucella bacteria genus (Montalvo et al., 2010).

In Peru, 15 cases of GBS were reported between April and May of 2018 in Trujillo, northern Peru, during summer time, activating a national epidemiological alert declared by the Ministry of Health. All cases were put on immunoglobulin G and managed in the intensive care unit at a regional Hospital. Blood samples were taken in all cases for both environmental exposure and DNA extraction for further genetic analysis.

This study determines the occurrence of polymorphisms in *IL‐17*, *ICAM1*, and *CD1* in GBS cases with a medical history of enteric respiratory and/or gastrointestinal infection and controls.

## 2 PATIENTS AND METHODS

### 2.1 Ethical approval

This study was approved by the ethics and research committee of Belen Hospital of Trujillo, northern Peru. A written informed consent was obtained from all subjects prior to recruitment for the study.

### 2.2 Cases and controls

Nine patients with GBS (seven men and two women, age: 52–65 years) followed at a regional hospital in northern Peru were enrolled in the study during the outbreak of GBS occurred in May 2018. Eleven healthy subjects (seven women and four men, age: 27–74 years) were randomly selected as controls from the same geographical area of residence. A total of 3 ml of blood was obtained from peripheral veins in all subjects.

### 2.3 Isolation of DNA and genotyping of *IL‐17*, *ICAM1*, and *CD1*

One hundred microliters of peripheral blood was used to extract DNA using QIAamp DNA Blood Kit Mini (Qiagen, CA, USA) and INBIOMag Genomic DNA Kit (INBIOMEDIC, Peru). According to the literature and genotypes location reported, the following fragments were selected: *IL‐17*, *ICAM1*, and *CD1* (2 fragments, found in exon 2). Specific primers were designed for each DNA fragment (Table 1) and the fragments were PCR amplified using Taq PCR Master Mix Kit (Qiagen, CA, USA). PCR products were purified and sequenced by Sanger technique in Macrogen (Soul Korea).

**Table 1:** Primers used in the genetic analysis of Guillain–Barre Syndrome

Gen	Primers (5′→3′)
*CD1A*	AGACGGGCTCAAGGAGCCTC
	TTCAAACTGCAATTCATGGGC
*CD1E*	GAGGAGCAGCTGTCCTTCCG
	ATTGACCAGCAGAAGCTTGC
*IL‐17*	GTTGTACAGGCCCAGTGTAG
	GGATATGCACCTCTTACTGC
*ICAM1*	CCGTGGTCTGTTCCCTGTAC
	GAAGGAGTCGTTGCCATAGG

### 2.4 Genetic analysis

The DNA sequence data were processed using Geneious R11 software (Biomatters Ltd.). The polymorphisms in *IL‐17* [NM_052872.3:c.377A>G (p.Glu126Gly)], *ICAM1* [NM_000201.3:c.721G>A (P.Gly241Arg)], *CD1A* [NC_000001.11:g.158248722 C>G (p.Thr13Ile)], and *CD1E* [NC_000001.11:g.158354032G>A (p.Glu79Arg)] genotypes were evaluated. The sequences obtained from *IL‐17*, *ICAM1*, and *CD1* were compared with sequences reported in previous research and/or global database.

### 2.5 Statistical analysis

Polymorphisms of *IL‐17*, *ICAM1*, and *CD1* were listed by frequency and percentage. Exploratory analysis comparing frequency of polymorphisms between cases and controls were performed by the chi square test and logistic regression models. Results were considered significant if *p* < .05. Statistical analysis was performed by SATA version 15.0 (Illinois, USA).

## 3 RESULTS

A total of nine cases (seven males, 77.8%) analyzed (Table 2) were diagnosed as GBS of atypical presentation. Eight patients reported some type of symptomatology 8 weeks before the onset of paralysis such as respiratory, gastrointestinal infections, nonpurulent conjunctivitis, joint and head pain. Only one reported a trip to Virú province days before the paralysis.

**Table 2:** Bivariate analysis of factors associated with the diagnosis of Guillain–Barre Syndrome

Variables	Guillain–Barre Syndrome		*p*
	Yes (*n* = 9)	No (*n* = 11)	
	*n*(%)	*n*(%)	
Sex			.080^a^
Male	7 (63.6)	4 (36.4)	
Women	2 (22.2)	7 (77.8)	
Age	55 (52–65)*	44 (34–63)*	.087^b^
BMI			.013^a^
Malnourished (<18)	1 (100.0)	0 (0.0)	
Normal (18–25)	2 (16.7)	10 (83.3)	
Overweight (>25)	4 (80.0)	1 (20.0)	
*rs2269715 (CD1A)*			.361^a^
01/01 (CC)	6 (37.5)	10 (62.5)	
01/02 (GC)	2 (66.7)	1 (33.3)	
02/02 (GG)	1 (100.0)	0 (0.0)	
*rs2269714 (CD1A)*			.421^a^
01/01 (CC)	6 (37.5)	10 (62.5)	
01/02 (CT)	2 (100.0)	0 (0.00)	
02/02 (TT)	1 (50.0)	1 (50.0)	
*rs1065457 (CD1E)*			.816^a^
01/01 (AA)	1 (33.3)	2 (66.7)	
01/02 (AG)	6 (42.9)	8 (57.1)	
02/02 (GG)	2 (66.7)	1 (33.3)	
*rs1799969 (ICAM1)*			.040^a^
01/01 (GG)	6 (71.4)	2 (28.6)	
01/02 (GA)	3 (25.0)	9 (75.0)	
02/02 (AA)	0 (0.0)	0 (0.0)	

Note

*p *value from statistical test: ^a^Fisher’s exact. ^b^Mann–Whitney.

Abbreviation: BMI, body mass index.

* Median (Q1–Q3).

All nine cases experienced muscle weakness, six of them also complained of pain, three cases presented ataxia, two cases had cranial nerves compromise and three cases presented symmetric paralysis. Autonomic disturbances, urinary dysfunction, sinus tachycardia, and arrhythmia, were demonstrated in each case.

Among controls and GBS cases, *IL‐17* is monomorphic in 01/01 genotype. Table 3 shows the frequencies of *CD1A*, *CD1E*, and *ICAM1* alleles and genotypes in controls and patients with GBS. *CD1A* is biallelic. Allele 01 is more frequent in both controls and patients with GBS. CD1A*02/02 genotype is not represented in controls and is present in only one of nine patients with GBS. CD1A*01/01 genotype is slightly more frequent in control patients compared with GBS.

**Table 3:** Genotype and frequency of alleles for *CD1A*, *CD1E*, *IL‐17*, and *ICAM1* in GBS patients and controls

	Genotype			% of allele		% of persons	
	Persons number (%)			Frequency		Positive for alleles	
	01/01	01/02	02/02	01	02	01	02
***CD1A***							
*rs2269715*							
GBS	6 (67%)	2 (22%)	1 (11%)	82	18	89	33
Control	10 (91%)	1 (9%)	0 (0%)	95	5	100	9
*rs2269714*							
GBS	6 (67%)	2 (22%)	1 (11%)	82	18	89	33
Control	10 (91%)	0 (0%)	1 (9%)	91	9	91	9
***CD1E***							
GBS	1 (11%)	6 (67%)	2 (22%)	44	56	78	89
Control	2 (18%)	8 (73%)	1 (9%)	55	45	91	82
***IL−17***							
GBS	9 (100%)	—	—	100	—	100	—
Control	11 (100%)	—	—	100	—	100	—
***ICAM1***							
GBS	6 (67%)	3 (33%)	0 (00%)	83	17	100	33
Control	0 (0%)	2 (18%)	9 (82%)	9	91	18	100

Note

*CD1A* [NC_000001.11:g.158248722 C>G (p.Thr13Ile)]

*CD1E* [NC_000001.11:g.158354032G>A (p.Glu79Arg)]

*IL‐17* [NM_052872.3:c.377A>G (p.Glu126Gly)]

*ICAM1* [NM_000201.3:c.721G>A (P.Gly241Arg)]

*CD1E* has two alleles with approximately the same frequency in controls and patients. (Table 3). CD1E*01/02 genotype is more frequent in both controls and patients with GBS. *ICAM1* is biallelic (Table 3). Allele 01 is more frequent in patients with GBS than in controls. ICAM1*01/01 genotype is not represented in controls and is present in six of nine patients with GBS. ICAM1*02/02 genotype is not represented in GBS patients and is present in nine of 11 control patients.

According to a first bivariate analysis (Table 2), the risk of being diagnosed with GBS in people with *ICAM1* GA genotype is lower compared to people with *ICAM1* GG genotype and this difference is statistically significant (*p* = .040). No statistically significant differences were found between groups of patients studied according to GBS diagnosis and other covariates analyzed: sex, age, genotypes *CD1A*, *CD1E*, *IL‐17* (*p* > .05).

According to regression analysis, *ICAM1* genotype and BMI variables contribute statistically to association under study (Table 4); Thus, the risk (OR) of being diagnosed with GBS in people with *ICAM1* GA genotype is about one‐third (33%) compared with people with *ICAM1* GG genotype (95% CI: 0.11–0.99; *p* = .047). The rest of covariates with exception of BMI do not contribute statistically significant to the association under study.

**Table 4:** Regression analysis of factors associated with the diagnosis of Guillain–Barre Syndrome

Features	Bivariate analysis		
	OR	95% CI	*p*
Sex			
Women	Ref.		
Male	2.86	0.75–9.17	.122
Age (years)	1.03	0.99–1.07	.136
BMI			
Normal	Ref.		
Malnourished	6.0	1.63–22.06	.007
Overweight	4.8	1.21–19.04	.026
*rs2269715 (CD1A)*			
01/01 (CC)	Ref.		
01/02 (CG)	1.78	0.62–5.06	.281
02/02 (GG)	2.67	1.39–5.10	.003
*rs2269714 (CD1A)*			
01/01 (CC)	Ref.		
01/02 (CT)	2.67	1.39–5.10	.003
02/02 (TT)	1.33	0.28–6.36	.718
*rs1065457 (CD1E)*			
01/01 (AA)	Ref.		
01/02 (AG)	1.29	0.22–7.44	.779
02/02 (GG)	2	0.32–12.54	.459
*rs1799969 (ICAM1)*			
01/01 (GG)	Ref.		
01/02 (GA)	0.33	0.11–0.99	.047

Note

*p* value from statistical test: Logistic regression.

Abbreviation: BMI, body mass index.

## 4 DISCUSSION

This is the first analysis of *IL‐17*, *ICAM1*, and *CD1* polymorphisms in Peruvian patients with GBS and healthy controls. Significant differences in the frequency of *ICAM1* SNPs were observed between patients with GBS and healthy controls, implying that *ICAM1* polymorphisms do influence susceptibility to GBS in the Peruvian population. Additionally, no genetic associations were observed between *IL‐17* and *CD1* polymorphisms and GBS susceptibility.

Members of the CD1 family are key players in the immune response to glycolipids and may be involved in the GBS pathogenesis, especially in patients with history of *C.* *jejuni* infections and antiganglioside antibodies (Caporale et al., 2006; De Libero et al., 2005). SNPs in *CD1B* (OMIM #188360), *CD1C* (OMIM #188340), and *CD1D* (OMIM #188410) were not determined in the current study because these are very rare and/or silent (Mirones, Oteo, Parra‐Cuadrado, & Martinez‐Naves, 2000).

*CD1A* and *CD1E* are biallelic in exon 2 (Han, Hannick, DiBrino, & Robinson, 1999). *CD1E* is the most polymorphic gene and reports variants in exon 3 also (Han et al., 1999; Mirones et al., 2000). In our study, no significant differences were found in the SNPs frequency of *CD1A* and *CD1E* between GBS patients and healthy controls, which indicates that these genetic polymorphisms do not influence the susceptibility to GBS development in the population studied. (Table 3). In addition, there was no genetic association with clinical outcome in GBS patients. These results do not support the hypothesis that *CD1A* and *CD1E* influence GBS risk as it was raised in a previous study that was based on an Italian cohort of GBS patients (Caporale et al., 2006). It is likely that this discrepancy is caused by differences in patient populations, although both had an ethnic origin, with a similar distribution of polymorphic frequencies in *CD1E*, but different in *CD1A*. On the other hand, it should be noted that the absence of association with polymorphisms of *CD1* does not exclude the possibility that *CD1* molecules play an important role in GBS pathogenesis. More research is needed to determine if it is *CD1* molecules or pathways subsequent to *CD1*, those that participate in process of glycolipids antigenic presentation in GBS.

Many association studies have reported that *IL‐17* polymorphisms predispose to autoimmune and inflammatory diseases (Arisawa et al., 2008; Jang et al., 2008; Kawaguchi et al., 2006; Seiderer et al., 2008). However, some *IL‐17F* polymorphisms (Glu126Gly and His161Arg) may not be significantly associated with autoimmune diseases (Paradowska‐Gorycka et al., 2010). There are no data reported in the context of *IL‐17F* polymorphism with GBS. The importance of *IL‐17F* polymorphism in GBS is still largely unknown. In our study, it was observed that *IL‐17* is monomorphic in 01/01 genotype for patients with GBS and controls.

Increased expression of *ICAM1* has been demonstrated in endothelial cells, microglia, and astrocytes in patients with multiple sclerosis (Carrithers, Visintin, Kang, & Janeway, 2000; Mycko, Kwinkowski, Tronczynska, Szymanska, & Selmaj, 1998). We have observed significant protection association with GBS in people with heterozygous GA genotype in *ICAM1* (241Gly/Arg) (*p* < .047). These results do not support the association hypothesis of significant risk of heterozygous *ICAM1* genotype (241Gly/Arg) in GBS as it was raised in a previous control case study in India with GBS (Table 5). Here, we can see other associated diseases for *ICAM1* G241R polymorphisms studies. Differences can also be explained in part by statistical analysis methods (Bang, Zaykin, & Mazumdar, 2007; Caporale et al., 2006).

**Table 5:** Associated diseases of eight eligible studies for *ICAM1* G241R polymorphisms analysis

Diseases	OR	*p*	Population	Reference
Guillain–Barre syndrome	4.14	<.001	Indian	Kharwar et al. (2017)
Multiple sclerosis	0.64	<.200	Polish Caucasian	Mycko et al. (1998)
Cancer	2.03	<.010	Asian‐European‐American	Cheng and Liang (2015)
Cancer	1.95	<.010	European	Cheng and Liang (2015)
Fuchs uveitis	3.3	.012	Italian	Cimino et al. (2010)
Schizophrenia	1.14	.771	German Caucasian	Riedel et al. (2003)
Ischemic stroke	1.82	.001	ARIC Study (white)	Volcik, Ballantyne, Hoogeveen, Folsom, and Boerwinkle (2010)
Ischemic stroke	1.49	.300	ARIC Study (black)	Volcik et al. (2010)
Guillain–Barre syndrome	0.33	.047	Nor‐Peruvian	This study

*Abbreviation*: ARIC: Atherosclerosis Risk in Communities

The study number and sample size were limited, which may affect the reliability of the results. As well as, the differences may be explained by genetic diversities, different risk factors in life styles, and the exposure to different environmental factors. In conclusion, *ICAM1* polymorphisms might be considered as potential genetic markers of GBS susceptibility after studies with larger sample size and further validation in ethnically different populations.

## CONFLICT OF INTEREST

The authors declared no conflict of interest.
